# Supplementary material for: Gain-through-filtering enables tuneable frequency comb generation in passive optical resonators
Source: Nat Commun. 2019 Oct 3;10:4489. doi: 10.1038/s41467-019-12375-3 (PMC6776525; doi:10.1038/s41467-019-12375-3)
Supplement: Supplementary file 1 — Supplementary Information [file 41467_2019_12375_MOESM1_ESM.pdf]

Supplementary Information for:

Gain-through-filtering enables tuneable frequency comb generation in passive optical resonators

Bessin et al.

Supplementary Information for: Gain-through-filtering enables tuneable frequency comb generation in passive optical resonators

Florent Bessin[1], Auro M. Perego[2,*], Kestutis Staliunas[3,4], Sergei K. Turitsyn[2,5], Alexandre Kudlinski[1], Matteo Conforti[1], and Arnaud Mussot[1]

[1]Univ. Lille, CNRS, UMR 8523-PhLAM Physique des Lasers Atomes et Molcules, F-59000 Lille, France

[2]Aston Institute of Photonics Technologies, Aston University, Birmingham, B4 7ET, UK

[3]Institució Catalana de Recerca i Estudis Avançats, Pg. Lluis Companys 23, 08010, Barcelona, Spain

[4]Departament de Física, Universitat Politècnica de Catalunya, 08222, Terrassa, Spain

[5]Novosibirsk State University, Novosibirsk, 630090, Russia

*corresponding author: a.perego1@aston.ac.uk

SUPPLEMENTARY NOTE 1: DETAILS OF EXPERIMENTAL MEASUREMENTS

A detailed sketch of the experimental setup is depicted in Supplementary Fig. 1. It consists of a passive fibre cavity from a 102 m long dispersion shifted fibre (DSF) ($\beta_2^{\mathrm{DSF}} = 0.5$ ps^2/km at the pump wavelength, $\gamma = 2.5$/W/km), an isolator and a fibre Bragg grating (FBG) acting as a notch filter in transmission and a 90/10 coupler. The total cavity length is 104.2 m (coupler+fibre). The reflected light by the FBG is blocked by the isolator. The cavity is driven by a train of square shaped pulses of 1.5 ns duration. The configuration allows to avoid Brillouin scattering and to get high peak power pulses to trigger the parametric processes. Pulses are generated from a continuous wave (CW) laser at 1545 nm intensity modulated by an electro-optical modulator (EOM). The repetition rate is set to match with the repetition rate of the cavity (1.96 MHz) to get one pulse per roundtrip. Pulses are then amplified by an erbium-doped fibre amplifier (EDFA) and pass through a thin filter (BPF, 1 nm width) to remove amplified spontaneous emission (ASE) in excess. At the output of EOM1, a weak part of the pulse train is collected by means of a 80/20 coupler, and then combined with the powerful pulse train using a 50/50 polarisation maintaining coupler. The weak and the strong pulse trains are cross polarised and are interleaved in the time domain to not interact. Both trains are then launched in the cavity in the anti-clockwise direction. At the cavity output, the spectrum is analysed with an optical spectrum analyser (OSA) and the weak pulse train is isolated from the strongest one in two stages. First by using a polarisation beam splitter and then with EOM2 that is synchronised with EOM1. Finally, the weakest signal experiences an isolation of more than 60 dB from the strong one and can be used as a reference signal to stabilise the cavity. We used a feedback loop system (proportional-intergral-derivative-controller) to keep both the driving frequency of the pump laser and the overall cavity length stable. The setup is very similar to schemes implemented in Supplementary Refs. [1–3] except that the probe signal is co-propagating because of the use of an isolator inside the cavity. All spectra shown in this paper were recorded from this stabilisation scheme because in this way the cavity detuning can be precisely determined [1–3] (Oscillo. 1). In the time domain, a commercial time lens system [4] (Picoluz ultrafast temporal magnifier, Thorlabs) connected to a high band-pass oscilloscope (Oscillo. 2) and photo-detector (70 GHz bandpass each) is used to get a temporal resolution of 300 fs over 50 ps with a magnification factor of 57. This resolution is good enough to analyse the temporal patterns associated with the modulation instability whose period of modulation is about 1/588 GHz$\simeq$1.7 ps. The time lens system is pumped by an ultra-stable laser (MENLO) at 100 MHz repetition rate. Note that it is not necessary to synchronise the time lens pump with the pulse launched inside the cavity as we did not aim at performing roundtrip to roundtrip measurements. We analysed output traces when the cavity reached its steady state by recording a large set of data and by selecting those for which the time lens pump and the cavity output pulses temporally overlap. A single example is shown in Fig. 4 of the main paper, but many similar recordings were obtained which proves that the cavity had indeed reached its steady state. Excellent cavity stabilisation is required to get clean time traces. Thus, we slightly modified the experimental setup for these time measurements to improve the stabilisation accuracy. Rather than using an error signal co-propagating with the pump, we maximised the power of one of the parametric sidebands as in Supplementary Ref. [5]. We used a bandpass filter (BPF2, 1 nm bandpass at FWHM) to select the frequency of the sideband we wanted to destabilise. We fixed it exactly at the frequency shift we had in Fig. 2 of the main paper, 588 GHz. Hence, as the cavity detuning is automatically set by the system, we can fairly assume that it is close to the value measured with the

other stabilisation system, i.e. $\phi_0 = -0.04$ rad. The system is much more stable in this way and we have been able to record very clean temporal traces.

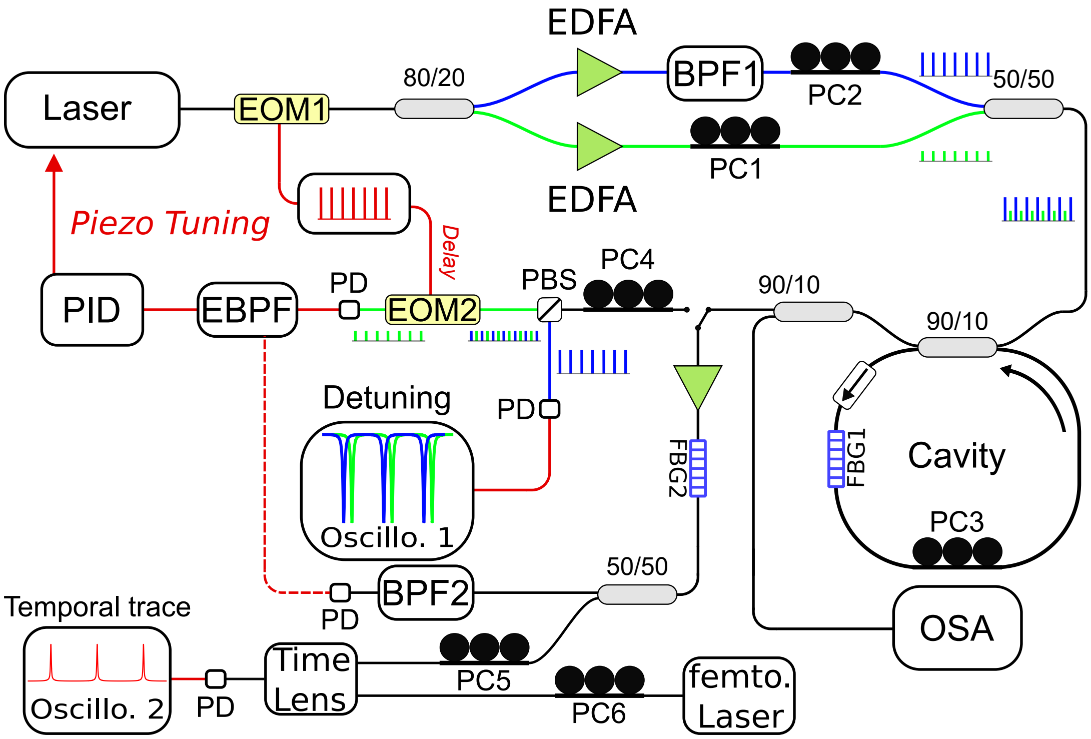

Supplmentary Fig. 1 Experimental setup. PC_{1-6}, polarisation controllers; STR, stretcher; PD, photo-detector; $EOM_{1,2}$, electro-optic modulators; OSA, optical spectrum analyser; EDFA, erbium-doped fibre amplifier; $FBG_{1,2}$, fibre Bragg grating; $BPF_{1,2}$, band-pass filters; PID, proportional-integral-derivate controller; EBPF, electronic band-pass filter.

SUPPLEMENTARY NOTE 2: TEMPORAL AND SPECTRAL EVOLUTION OF THE INTRACAVITY FIELD

In this section we report the evolution of the intracavity field (as a function of the roundtrip number) calculated form numerical solution of the Ikeda map, and corresponding to the output fields reported in Figs. 2 and 4 of the main paper. The parameters used for the simulations are reported in the Methods section of the Manuscript. Supplementary Fig. 2(a) shows the building of the frequency comb in time domain starting from an empty cavity. After a few roundtrips, the CW steady state of the cavity is reached. GTF mechanism amplifies the noise and produces an exponentially growing modulation. Due to the dissipative character of the resonator, the growth saturates towards a periodic train of pulses, which becomes stable after around 150 roundtrips. The negative group velocity of the pattern is caused by the odd part of the overall dispersion [1, 6], which is a combination of the third order dispersion of the fibre and the odd part of the filter phase. Supplementary Fig. 2(b) shows the evolution of the spectrum. At roundtrip 80 two symmetric sidebands at ±580GHz from the pump start to become visible and correspond to the peak of GTF gain. Then, several lines are generated through multiple four-wave mixing, until a stable comb is obtained after around 250 roundtrips. Figures 2(c-d) show a zoom on the dynamics along the fibre (inside the cavity) when a stable comb is reached (roundtrips 300 and 301 are showed). A significant reshaping in time domain is showed in Supplementary Fig. 2(c) from $z = 0$ to $z = L = 104.2$m (roundtrip 300). The field regains the initial shape after the injection of the pump and the action of the filter. The evolution during roundtrip 301, indexed by fibre length from L to $2L$ in our representation, is essentially identical. The power spectrum showed in Supplementary Fig. 2(d) does not show any significant modifications, meaning that the deformation seen in time domain are mainly caused by a different phase acquired by each spectral line during the propagation along the fibre.

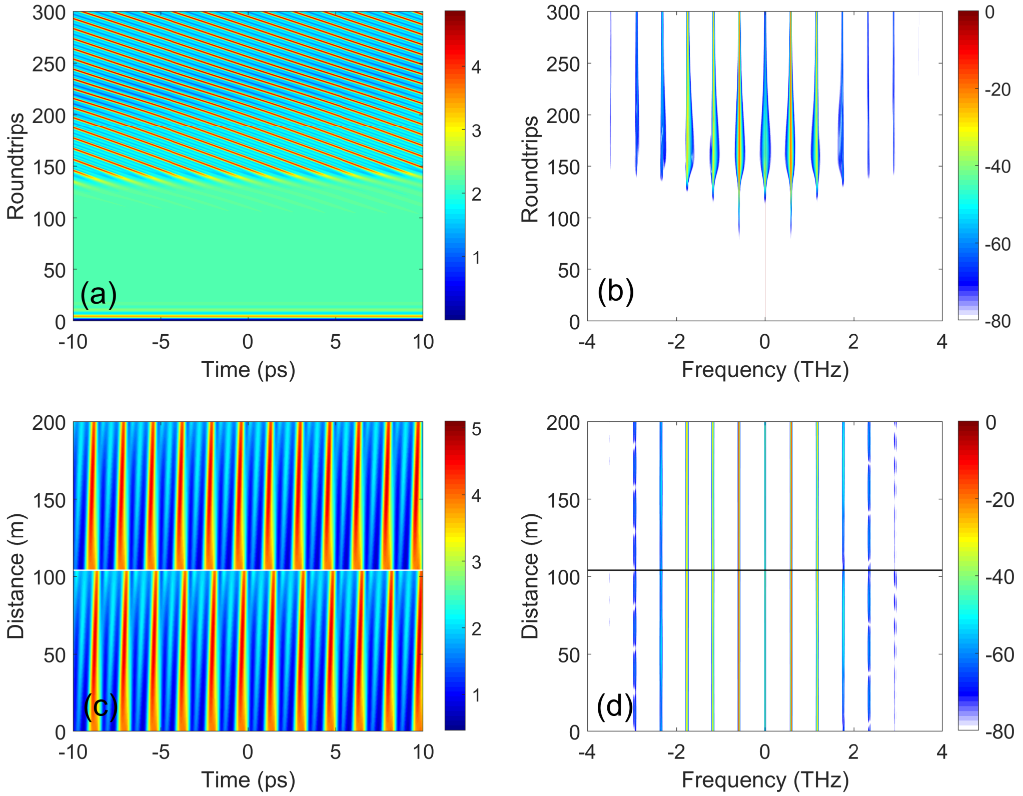

Supplementary Fig. 2 Temporal and spectral evolution of the intracavity field. Power (a) and spectrum (b) at the position of the coupler ($z = 0$) as a function of roundtrips. Intracavity field power (c) and spectrum (d) as a function of the position in the fibre for two successive roundtrips (300 and 301). The following parameters have been used: $\beta_2 = 0.5$ ps^2/km , $\beta_3 = 0.12$ ps^3/km, $\gamma = 2.5$ W^{-1}km^{-1}, $L = 104.2$ m, km^{-1}, $\rho^2 = 0.5940$ (cavity finesse $F = 12$) , $\theta^2 = 0.1$, $\phi_0 = -\arg[H(0)]$ (the measured total cavity detuning is zero), input power $P_{\mathrm{IN}} = 6.6$ W , frequency shift of the filter $\omega_{\mathrm{f}}/(2\pi) = 399$ GHz.

SUPPLEMENTARY NOTE 3: ANALYTICAL CALCULATION OF THE PARAMETRIC GAIN AND PHASE MATCHING CONDITION

Statement of the problem

The starting point of our theoretical treatment is the following Ikeda map in dimensional units

$$i\frac{\partial A_n}{\partial z} - \frac{\beta_2}{2}\frac{\partial^2 A_n}{\partial t^2} + \gamma|A_n|^2 A_n = 0, \quad 0 < z < L, \tag{1}$$

$$A_{n+1}(z=0,t) = \theta E_{in} + \rho e^{i\phi_0} A_n(z=L,t). \tag{2}$$

All the losses (except the filter induced ones) are lumped in ρ, so that $1-\rho^2$ measures the total power loss per roundtrip. ϕ_0 is the linear phase shift per roundtrip. Third order dispersion has been neglected because it does not affect the parametric gain. The filter located at the position $z = z_F$ acts in the following way:

$$A_n(z_F^+, t) = h(t) * A_n(z_F^-, t), \tag{3}$$

$$\hat{A}_n(z_F^+, \omega) = H(\omega)\,\hat{A}_n(z_F^-, \omega), \tag{4}$$

where $h(t)$ is the filter response (causality imposes $h(t) = 0$ if $t < 0$), $*$ denotes convolution and $H(\omega) = \hat{h}(\omega) = \int_{-\infty}^{+\infty} h(t)\exp[i\omega t]dt$ is the filter transfer function. For simplicity, the filter is assumed to be placed just before the coupler ($z_F = L$). This way, the boundary conditions and filter can be conveniently combined in the single equation:

$$A_{n+1}(z=0,t) = \theta E_{in} + \rho e^{i\phi_0} h(t) * A_n(z=L,t) \tag{5}$$

Steady state

We search for the stationary ($\partial_t = 0$ or equivalently $\omega = 0$) field inside the fibre as

$$A_n(z,t) = A_{\mathrm{s}} e^{i\gamma P z}, \quad P = |A_{\mathrm{s}}|^2.$$

The relation between the field circulating into the cavity and the pump is (for complex field and power)

$$A_{\mathrm{s}} = \frac{\theta}{1-\rho e^{i\phi} H(0)} E_{\mathrm{IN}}, \tag{6}$$

$$P_s = \frac{\theta^2}{1+\rho^2|H(0)|^2 - 2\rho|H(0)|\cos(\phi + \sphericalangle H(0))} P_{\mathrm{IN}} \tag{7}$$

where the total phase shift imposed by the cavity is $\phi = \phi_0 + \gamma P L$ while E_{IN} and P_{IN} denote the amplitude and the power of the pump field respectively.

Linear stability analysis

In order to study the stability of the steady state defined by Supplementary Eq. (6) we consider the following perturbation

$$A_n(z,t) = [\sqrt{P} + \eta(z,t)] e^{i\gamma P z}, \quad |\eta| \ll \sqrt{P}.$$

For simplicity we have assumed the intracavity field to be real (the phase can be accounted for in the pump field). By linearisation we obtain the equation governing the evolution of the perturbations:

$$i\eta_z - \frac{\beta_2}{2}\eta_{tt} + \gamma P(\eta + \eta^*) = 0.$$

We split perturbations in real and imaginary parts $\eta = a + ib$ ($a, b \in \mathbb{R}$), to get the following system in frequency domain:

$$\hat{a}_z(z,\omega) = -\frac{\beta_2\omega^2}{2}\hat{b}(z,\omega), \tag{8}$$

$$\hat{b}_z(z,\omega) = \left(\frac{\beta_2\omega^2}{2} + 2\gamma P\right)\hat{a}(z,\omega). \tag{9}$$

The solution of the system from $z = 0$ to $z = L$ gives the perturbations after one pass in the fibre as:

$$\begin{bmatrix} \hat{a}(L) \\ \hat{b}(L) \end{bmatrix} = \begin{bmatrix} \cos(kL) & -\frac{\beta\omega^2}{2k}\sin(kL) \\ \frac{2k}{\beta\omega^2}\sin(kL) & \cos(kL) \end{bmatrix} \begin{bmatrix} \hat{a}(0) \\ \hat{b}(0) \end{bmatrix}, \tag{10}$$

where $k^2 = \frac{\beta_2\omega^2}{2}\left(\frac{\beta_2\omega^2}{2} + 2\gamma P\right)$ describes the dispersion relation of the small harmonic perturbations which propagate on top of the stationary field.

The combined action of the filter and the coupler on the perturbations can be written as follows:

$$\begin{bmatrix} \hat{a}_{n+1}(0) \\ \hat{b}_{n+1}(0) \end{bmatrix} = \rho \begin{bmatrix} \cos\phi & -\sin\phi \\ \sin\phi & \cos\phi \end{bmatrix} \begin{bmatrix} H_{\mathrm{e}}(\omega) & -H_{\mathrm{o}}(\omega) \\ H_{\mathrm{o}}(\omega) & H_{\mathrm{e}}(\omega) \end{bmatrix} \begin{bmatrix} \hat{a}_n(L) \\ \hat{b}_n(L) \end{bmatrix}, \tag{11}$$

where we have defined the even and odd part of the transfer function as

$$H_{\mathrm{e}}(\omega) = \mathcal{F}\{\mathrm{Re}[h(t)]\} = \frac{1}{2}\big(H(\omega) + H^*(-\omega)\big),$$

$$H_{\mathrm{o}}(\omega) = \mathcal{F}\{\mathrm{Im}[h(t)]\} = \frac{1}{2i}\big(H(\omega) - H^*(-\omega)\big).$$

Note that the coupler and filter matrices commutes, as expected intuitively: for the stability analysis it doesn't matter if the filter is placed just before or just after the coupler.

By combining Supplementary Eqs. (10-11) we get the total effects accumulated by the perturbations over one roundtrip as

$$\begin{bmatrix} \hat{a}_{n+1}(0) \\ \hat{b}_{n+1}(0) \end{bmatrix} = M \begin{bmatrix} \hat{a}_{n}(0) \\ \hat{b}_{n}(0) \end{bmatrix}. \tag{12}$$

The eigenvalues of M reads as

$$\lambda_{1,2} = \frac{\Delta}{2} \pm \sqrt{\frac{\Delta^2}{4} - W} \tag{13}$$

where

$$W = \rho^2 \left(H_\mathrm{e}(\omega)^2 + H_\mathrm{o}(\omega)^2\right), \tag{14}$$
$$\Delta = \rho\left[2\cos(kL)\left(H_\mathrm{e}(\omega)\cos\phi - H_\mathrm{o}(\omega)\sin\phi\right) - \frac{\beta_2\omega^2 + 2\gamma P}{k}\sin(kL)\left(H_\mathrm{o}(\omega)\cos\phi + H_\mathrm{e}(\omega)\sin\phi\right)\right].$$

Whenever $|\lambda_{1,2}| > 1$ the CW solution, Supplementary Eq. (6), is unstable and the perturbation amplitude grows as $\exp[g(\omega)z]$, with MI gain $g(\omega) = \ln(\max\{|\lambda_1|, |\lambda_2|\})/L$.

The spectral response of the filter is taken here to be $H(\omega) = \exp[\alpha(\omega) + i\psi(\omega)]$, where $|H(\omega)| = 1 - R\exp(-(\omega - \omega_f)^4/(\sigma)^4)$ and the filter phase is computed from the Bode's magnitude-phase relation: $\psi(\omega) = \arg[H(\omega)] = -\mathcal{H}[\alpha(\omega)]$, being $\mathcal{H}[.]$ the Hilbert transform. By using $R = 0.96$, $\omega_f = 399 \times 2\pi$ rad/ns, $\sigma_f = 160 \times 2\pi$ rad/ns we get a satisfactory approximation of the filter used in the experiments (see Fig. 2 of the main paper).

Analytical estimation of comb tuneability and instability threshold

In order to provide a clearer understanding of the comb repetition rate tuneability and of the threshold of the parametric oscillation, we have plotted the maximum GTF power instability gain ($\max\{2g(\omega)\}$) obtained from Supplementary Eqs. (13-14) (see also Eq. (5) in Methods section of the main paper). Supplementary Fig. 3(a) shows the maximum gain versus the input pump power for three different filter frequency shifts, while keeping the remaining parameters constant and consistent with the experimental ones. The input power threshold for GTF instability is ≈ 1.25 W for both the filters shifted 300 and 400GHz from the pump, whereas is substantially higher (≈ 2.2 W) for the 500 GHz filter. As a general remark, the higher the frequency shift, the lower is the gain, as intuitively expected. By increasing the input power above the threshold, after a first monotonic increase, the gain saturates, which partially explains why stable combs are observed well above the instability threshold. For higher input powers (not considered here), the gain eventually rises again, but in this region other competing instabilities may appear [7] and the dynamics is expected to turn rapidly into chaos, as the cavity is driven far beyond the instability threshold. Supplementary

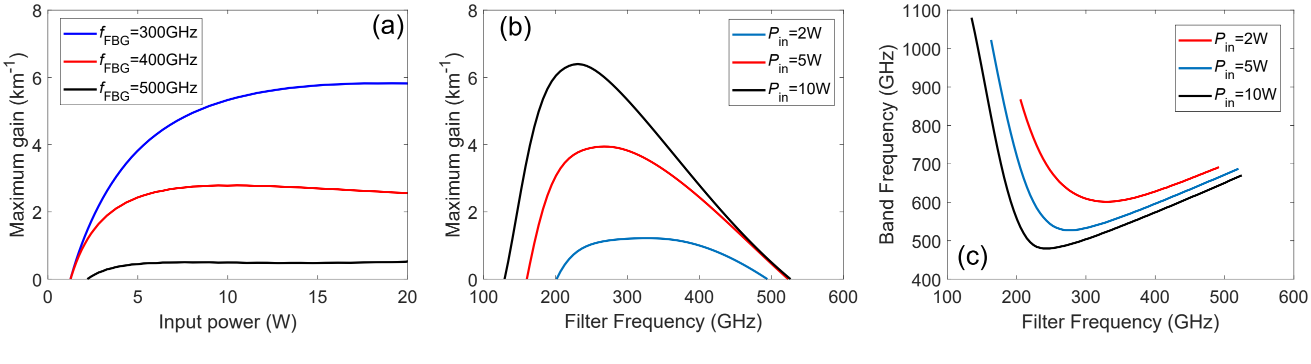

Supplementary Fig. 3 Gain dependence on system parameters. Theoretical maximum GTF gain as a function of input power (a) and frequency shift of the filter (b). The following parameters has been used: $\beta_2 = 0.5$ ps^2/km , $\beta_3 = 0.12$ ps^3/km, $\gamma = 2.5$ W^{-1}km^{-1}, $L = 104.2$ m, km^{-1}, $\rho^2 = 0.5940$ (cavity finesse $F = 12$) , $\theta^2 = 0.1$, $\phi_0 = -\arg[H(0)]$ (zero cavity detuning).

Fig. 3(b) shows the maximum gain versus filter frequency shift for three different input powers. As expected, the gain

decreases for large filter frequency and GTF instability is turned off for detunings higher than ≈ 500 GHz (for input powers in the order of 10 W). When the filter is moved close to the pump, the gain grows up to a maximum, then it starts to decrease. The filter considered here has a quite large stop-band (FWHM transmission ≈ 300 GHz), then for frequencies lower than ≈ 200 GHz it starts to affect the pump itself, reducing the nonlinear effects in the cavity. This eventually explains the low frequency cut-off for the GTF instability observed here. Supplementary Fig. 3(c) reports the frequency of the most unstable mode as a function of the filter frequency shift for three different input powers. For the example considered here, the comb repetition rate is tuneable in a range spanning 600 GHz ($480 - 1080$ GHz from the pump). In our experimental setup we could access only filter frequency shifts higher than 300 GHz due to the tuneability range of the laser. In this region, the repetition rate of the comb grows almost linearly as a function of the filter frequency (see Fig. 3(b) of the main paper). For filter frequency shift less that ≈ 300 GHz the pump itself is affected by the phase of the filter. Since in this example (and in the experiments) we are keeping the overall cavity detuning equal to zero, the linear phase shift ϕ_0 acquired during the propagation along the fibre gets bigger as the filter approaches the pump in order to compensate for the filter phase contribution to the pump. This fact substantially affects the cavity phase, pushing the phase matching towards higher frequencies (see Supplementary Fig. 5 below).

Approximations and phase matching condition

Supplementary Eqs. (13,14) give the exact parametric gain, however they do not allow for a straightforward physical interpretation. We then proceed to obtain an approximated formula. By exploring the parameters' space, we have noted that the position of the unstable bands is mainly fixed by the filter phase. Supplementary Fig. 4 shows the relative impact of the filter amplitude, phase and both combined on the GTF instability gain spectrum. The red curve, accounting for only $|H(\omega)|$, shows that the threshold of GTF instability is not reached. The most unstable band mimics the shape of the filter response, as expected for gain-through-loss mechanism. Additional bands are seen at around 1500 GHz, which correspond to boundary condition induced modulation instability [7]. The black curve accounts only for the filter phase, it gives a reasonable prediction of the frequency of the unstable bands and slightly overestimates the gain.

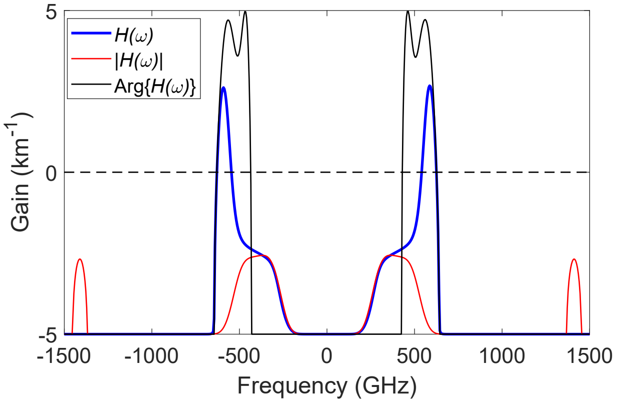

Supplementary Fig. 4 Instability gain $2g(\omega)$ calculated from Eqs. (13-14) revealing the effect of magnitude and phase of the filter transfer function. The following parameters has been used: $\beta_2 = 0.5$ ps^2/km , $\gamma = 2.5$ W^{-1}km^{-1}, $L = 104$ m, km^{-1}, $\rho^2 = 0.5940$ (cavity finesse $F = 12$) , $\theta^2 = 0.1$, $\phi_0 = -\arg[H(0)]$ (zero cavity detuning), $\omega_f/(2\pi) = 399$ GHz, intracavity power $P = 2.2$ W (input power $P_{IN} = 6.6$ W).

In order to predict position of the unstable band, we then assume the following form for the filter transfer function (unitary modulus)

$$H(\omega) = \exp[i\psi(\omega)]. \tag{15}$$

The even and odd part of the filter transfer function read as

$$H_\mathrm{e}(\omega) = e^{i\psi_\mathrm{o}(\omega)}\cos[\psi_\mathrm{e}(\omega)], \tag{16}$$

$$H_\mathrm{o}(\omega) = e^{i\psi_\mathrm{o}(\omega)}\sin[\psi_\mathrm{e}(\omega)], \tag{17}$$

where the even and odd part of the filter phase are defined as

$$\psi_\mathrm{e}(\omega) = \frac{\psi(\omega)+\psi(-\omega)}{2}, \tag{18}$$

$$\psi_\mathrm{o}(\omega) = \frac{\psi(\omega)-\psi(-\omega)}{2}. \tag{19}$$

The assumption of unitary modulus, permits to greatly simplify Supplementary Eqs. (14) as follows

$$W = \rho^2 e^{i2\psi_\mathrm{o}}, \tag{20}$$

$$\Delta = \rho e^{i\psi_\mathrm{o}}\left[2\cos(kL)\cos(\phi+\psi_\mathrm{e}) - \frac{\beta_2\omega^2+2\gamma P}{k}\sin(kL)\sin(\phi+\psi_\mathrm{e})\right] \triangleq e^{i\psi_\mathrm{o}}\tilde{\Delta},$$

which gives the following expression for the eigenvalues:

$$\lambda_{1,2} = e^{i\psi_\mathrm{o}}\left[\frac{\tilde{\Delta}}{2} \pm \sqrt{\frac{\tilde{\Delta}^2}{4}-\rho^2}\right]. \tag{21}$$

A part from the exponential factor, Supplementary Eq. (21) has been obtained before for the description of a standard cavity (i.e. without filter) [7, 8]. The exponential factor does not change the modulus of the eigenvalues, hence it does not affect the GTF gain. We have instability if $|\tilde{\Delta}| > 1+\rho^2$.

In order to find a phase matching relation, we expand the dispersion relation for the perturbations k as follows

$$k = \sqrt{\frac{\beta_2\omega^2}{2}\left(\frac{\beta_2\omega^2}{2}+2\gamma P\right)} \approx \frac{\beta_2\omega^2}{2}+\gamma P, \;\text{ if } |\omega| \gg \sqrt{\frac{\gamma P}{\beta_2}} \tag{22}$$

In this way we have

$$\tilde{\Delta} \approx 2\rho\cos[kL+\psi_\mathrm{e}(\omega)+\phi].$$

The potentially unstable frequencies maximise $|\tilde{\Delta}|$, and thus satisfy the following equation:

$$k(\omega)L+\phi+\psi_\mathrm{e}(\omega) = m\pi,\; m = 0, \pm 1, \ldots \tag{23}$$

The solutions of Supplementary Eq. (23) for $m \neq 0$ correspond to parametric resonances (PRs) induced by the periodic forcing represented by the injection of the pump at each roundtrip. For the range of parameters used in the experiments, these PRs bands appear, if they exist, at much higher frequencies with respect to GTF band.

We then concentrate on the $m = 0$ band and use the expansion (22), to get the following simple phase-matching relation:

$$\boxed{\frac{\beta_2\omega^2}{2}L + 2\gamma PL + \phi_0 + \psi_\mathrm{e}(\omega) = 0} \tag{24}$$

Supplementary Eq. (24) has a straightforward physical meaning: the phase acquired by the perturbations propagating in the fibre (kL) plus the total phase shift of the cavity (linear+nonlinear: $\phi = \phi_0 + \gamma P$) plus the even part of the phase of the filter ($\psi_e(\omega)$) must be zero to have parametric amplification. Equation (24) is a generalisation Eq.(8) of Ref. [7], including the dispersion induced by the filter.

In order to demonstrate the exactness of our treatment and the accuracy of the approximated phase-matching relation, we report in Supplementary Fig. 5 a numerical example. Supplementary Fig. 5(a) shows the magnitude and phase of the filter transfer function $H(\omega) = \exp[\alpha(\omega)+i\psi(\omega)]$ described above (see also Methods section in the Manuscript), which is shifted 399 GHz from the pump. Supplementary Fig. 5(b) shows the phase-matching curve from Supplementary Eq. (24) (dashed red curve), together with the different terms composing the equation: cavity-induced phase (solid blue curve) and even part of filter phase (solid green curve). Supplementary Fig. 5(c) shows

the gain spectrum after 50 roundtrips obtained from theory [Supplementary Eqs. (13-14), solid blue curve] and numerical solution of Ikeda map Supplementary Eqs. (1-5)(dashed red curve). The following parameters have been used: $\beta_2 = 0.5$ ps^2/km , $\beta_3 = 0.12$ ps^3/km, $\gamma = 2.5$ W^{-1}km^{-1}, $L = 104.2$ m, km^{-1}, $\rho^2 = 0.5940$ (cavity finesse $F = 12$) , $\theta^2 = 0.1$, $\phi_0 = -\arg[H(0)]$ (the measured total cavity detuning is zero), intracavity power $P = 2.2$ W, which corresponds to input power $P_{\mathrm{IN}} = 6.6$ W . The black dashed vertical line in Supplementary Figs. 5(a-c) denotes the phase matching frequency from Supplementary Eq. (24). From Supplementary Fig. 5(a) we can note that the phase matching frequency is very different from the centre of the filter, as it is the case for the standard gain-through-loss where filter dispersion does not play any significant role [9]. From Supplementary Fig. 5(c) we can appreciate that (i) numerical solution of Ikeda map agrees perfectly with the calculated MI gain and (ii) the frequency position of the maximum gain from theory and numerical simulation is well approximated by the simple phase matching formula Supplementary Eq.(24).

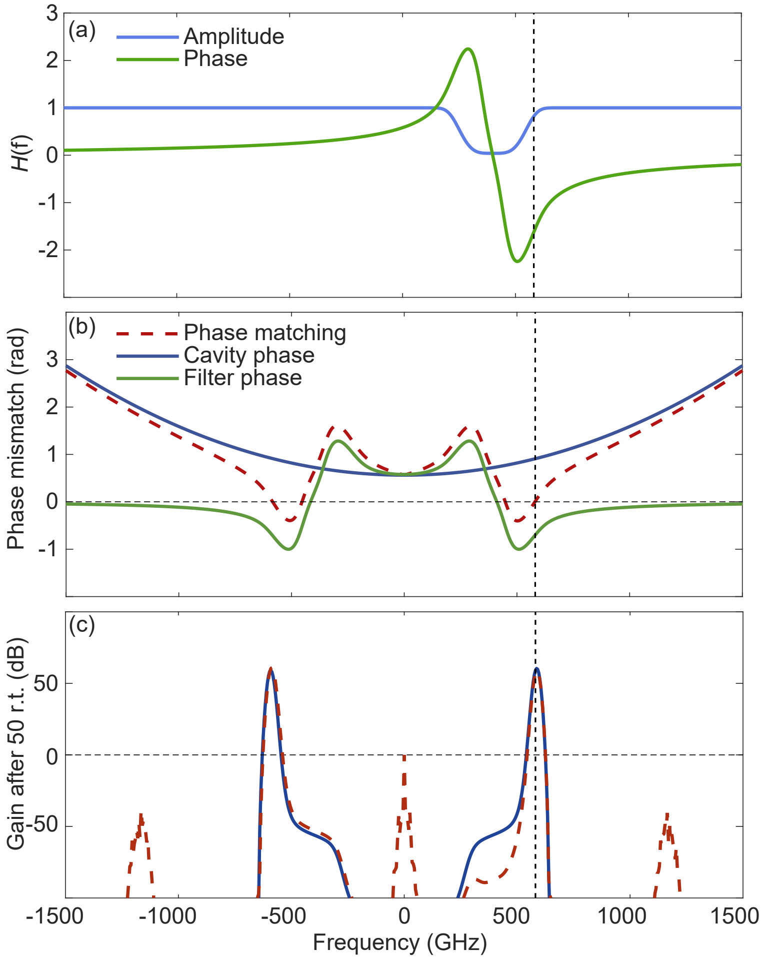

Supplementary Fig. 5 Cavity mismatch and gain spectrum. (a) Amplitude and phase of the filter with supergaussian shape. (b) Phase-matching curve from Eq. (24) (dashed red curve). The different terms composing the equation are also showed: cavity-induced phase (solid blue curve) and even part of filter phase (solid green curve). Gain spectrum after 50 roundtrips from theory [Eqs. (13-14), solid blue curve] and numerical solution of Ikeda map (dashed red curve).

SUPPLEMENTARY REFERENCES

[1] Mussot, A., Louvergneaux, E., Akhmediev, N., Reynaud, F., Delage, L., Taki, M., Optical Fiber Systems Are Convectively Unstable, *Phys. Rev. Lett.* **101**, 113904 (2008)
[2] Coen, S., Haelterman, M., Emplit, P., Delage, L., Simohamed, L. M., and Reynaud, F. Experimental investigation of the dynamics of a stabilized nonlinear fiber ring resonator, *JOSA B* **15**, 2283 (1998)
[3] Copie, F., Conforti, M., Kudlinski, A., Mussot, A., and Trillo, S. Competing Turing and Faraday Instabilities in Longitudinally Modulated Passive Resonators, *Phys. Rev. Lett.* **116**, 143901 (2016)
[4] Salem, R., Foster, M. A., and Gaeta, A. L. Application of space-time duality to ultrahigh-speed optical signal processing, *Adv. Opt. Photonics* **5**, 274 (2013)
[5] Coen, S., Haelterman, M., Continuous-wave ultrahigh-repetition-rate pulse-train generation through modulational instability in a passive fiber cavity, *Opt. Lett.* **26**, 3941, (2001)
[6] Leo, F., Mussot, A., Kockaert, P., Emplit, P., Haelterman, M., Taki, M. Nonlinear Symmetry Breaking Induced by Third-Order Dispersion in Optical Fiber Cavities, *Phys. Rev. Lett.* **110**, 104103, (2013)
[7] Coen, S., and Haelterman, M. Modulational Instability Induced by Cavity Boundary Conditions in a Normally Dispersive Optical Fiber, *Phys. Rev. Lett.* **79**, 4139-4142 (1997)
[8] Conforti, M., Copie, F., Mussot, A., Kudlinski, A., and Trillo, S. Parametric instabilities in modulated fiber ring cavities, *Opt. Lett.* **41**, 5027 (2016)
[9] Perego, A. M., Turitsyn, S. K. & Staliunas, K. Gain through losses in nonlinear optics, *Light: Science & Applications* **7**, 43 (2018)
